# Challenges associated with test dose pharmacokinetic predictions of high dose melphalan exposure in patients with multiple myeloma

**DOI:** 10.1007/s00228-022-03396-x

**Published:** 2022-10-07

**Authors:** Christa Ellen Nath, Andrew Grigg, Sebastian P. A. Rosser, Jane Estell, Elizabeth Newman, Campbell Tiley, Sundra Ramanathan, Shir Jing Ho, Stephen Larsen, John Gibson, Peter Presgrave, Peter John Shaw, Judith Trotman

**Affiliations:** 1 Biochemistry Department, The Children’s Hospital at Westmead, Westmead, Australia; 2 Cancer Centre for Children, The Children’s Hospital at Westmead, Westmead, Australia; 3 Clinical Haematology Department, Austin Hospital, Heidelberg, Australia; 4 Haematology Department, Concord Repatriation General Hospital, Concord, Australia; 5 Haematology Department, Gosford Hospital, Gosford, Australia; 6 Haematology Department, St George Hospital, Kogarah, Australia; 7 Haematology Department, Royal Prince Alfred Hospital, Camperdown, Australia; 8 Haematology Department, Wollongong Hospital, Wollongong, Australia; 9 Faculty of Health and Medicine, The University of Sydney, Camperdown, Australia; 10 The University of New South Wales, Kensington, Australia

**Keywords:** Melphalan, Test dose, Pharmacokinetic predictions, Myeloma

## Abstract

**Aim:**

To evaluate the accuracy of melphalan test dose pharmacokinetic (PK) predictions of the subsequent high dose (HDM) area under the concentration-versus-time curve (AUC) and to identify sources of prediction error (PE).

**Methods:**

A prospective multicentre PK study was conducted in 40 myeloma patients of median age 60 (range:35–71) years using a 20 mg/m^2^ test dose administered 1–3 days prior to HDM (predominantly 180 mg/m^2^). PK data were collected post the test and high doses to compare predicted versus actual AUCs determined using the trapezoidal rule. Test and high dose infusion concentration, volume and duration and the time from preparation to infusion were compared using the paired Wilcoxin rank sign test. The impact of Melphalan administration parameters on PE was evaluated using the Mann–Whitney test. The predictive capacity of a previously published population PK (PopPK) model was also examined.

**Results:**

Predicted HDM AUC was within 15% of the observed values in only 63% of patients when analysed using the trapezoidal rule and 70% of patients using PopPK. Test dose infusion concentration, volume, duration and time from preparation to infusion were significantly lower than for HDM (*p* < 0.005). Test dose administration within 15 min of reconstitution (*n* = 5) was associated with significantly lower PE than administration times of 16–60 min (*n* = 22), *p* < 0.05. Test and HDM infusion concentrations were lower in patients with large PE (> ± 15%), but the differences were not significant (*p* = 0.078, 0.228, respectively).

**Conclusion:**

Test dose PK has the potential to predict subsequent HDM exposure to achieve a target AUC once melphalan administration parameters are optimised to account for stability issues in the formulation.

**Supplementary Information:**

The online version contains supplementary material available at 10.1007/s00228-022-03396-x.

## Introduction

High-dose melphalan (HDM) followed by autologous stem cell transplantation (ASCT) has a well-established role in the treatment of patients with myeloma [1–4], and the progression-free survival benefit is still demonstrable in the era of modern combination therapies for remission induction [5–7].

HDM is conventionally administered using a body surface area (BSA)–based dose of 200 mg/m^2^, with reductions recommended in patients with impaired renal function [8, 9] which is consistent with renal excretion being the primary elimination route for melphalan [10, 11]. Historically, obese patients also received a reduced melphalan dose [12] but limiting the dose in obese patients, such as using an alternative weight descriptor in the BSA equation instead of actual weight, results in low HDM exposure, particularly in patients with good renal function [13]. These HDM dosing practices resulted in a fivefold variation in HDM exposure ranging from 5 to 25 mg/L.h for doses ranging from 115 to 216 mg/m^2^ [14]. Creatinine clearance, fat-free mass and haematocrit were identified as significant predictors of plasma melphalan clearance [14]. In this patient cohort, who were treated prior to widespread use of biological therapies, a high area under the concentration–time curve (AUC) above the median of 12.8 mg/L.h was associated with improved overall survival (8.5 years vs. 5.4 years, HR 0.4, *p* < 0.001) [15].

Since BSA-based melphalan dosing does not address the inter-patient variation in exposure [14], there is a need for additional dosing strategies for clinicians to optimise melphalan dose and exposure for individual patients. HDM is most commonly administered as a single high dose, often in an outpatient setting, or much less commonly, as two 100 mg/m^2^ doses 24h apart [16]. Pharmacokinetic (PK)-guided dose determination from an up to tenfold lower test dose has been previously examined [17–20], but the accuracy of test dose predictions of high dose exposure is highly variable. The application of the trapezoidal rule in early investigations of test dose melphalan PK concentrations to accurately (within 15%) predict the AUC of 1 to tenfold higher doses was successful in 63% (5/8) of patients when experimental conditions were uncontrolled but improved to 90% (9/10) of patients when conditions were tightly controlled with respect to drug administration (5 min infusion time) and blood sampling times, use of concomitant medication (suppression of frusemide) and use of experienced nursing staff, with the same nurse administering HDM and collecting PK blood samples for both the test and high doses [17]. The accuracy of the PK predictions has not improved in more recent studies conducted in 5 adults [19] and 24 children [20] in which predictions of AUC for a higher melphalan dose (140 to 200 mg/m^2^) from a test dose were within 15% observed values in 20% and 45% of patients, respectively.

Melphalan undergoes rapid spontaneous chemical hydrolysis in water and urine [11], and the melphalan infusion for administration also has limited stability, with nearly 1% label strength melphalan hydrolysing every 10 min [21]. Recent data on melphalan stability has demonstrated that, at room temperature, 4 mg/ml concentrations of melphalan admixture in 0.9% sodium chloride are stable for up to 8 h, whilst 0.5 and 2 mg/ml concentrations have limited stability of less than 1 h and 2 h, respectively [22]. Therefore the concentration and final volume of the melphalan infusion solution as well as the time from melphalan preparation to infusion end might also be expected to impact on AUC prediction accuracy from a test dose. Additional factors that are expected to impact on the feasibility and accuracy of the test dose strategy includes sample stability and transportation of sample from remote sites to the central laboratory and time-sensitive result reporting to allow timely administration of the high dose. Test dose prediction accuracy might also be impacted by the pharmacokinetic analysis method used to generate AUC. The use of the trapezoidal rule will provide an exact determination of AUC based on the measured concentration data, and these results will therefore highlight issues related to medication stability. Population pharmacokinetic (PopPK) modelling with Bayesian forecasting will provide an estimate based not only on the measured drug concentrations, but also on the components of the structural pharmacokinetic model, such as clearance and volume of distribution, the covariates which may impact them, such as renal function and body size, and also estimates of random error [23, 24] and can be expected to minimise issues related to medication stability resulting in improved prediction accuracy. The primary aims of this study were to evaluate (a) the feasibility of using a test dose of melphalan to provide PK data across multiple sites remote to the central laboratory and (b) to assess the accuracy of this test dose in predicting actual AUC after a subsequent dose of HDM and identify factors impacting on this accuracy, particularly, those related to medication stability. A secondary aim was to compare the prediction accuracy of AUC determined using the trapezoidal rule with that determined using the original PopPK model [14], which will also be externally validated as part of this study.

## Materials and methods

### Study design

This was a prospective, multi-centre, pilot study to evaluate the accuracy of actual HDM AUC attainment using PK prediction from a smaller test dose in 44 adult patients with myeloma. The characteristics of the study population are summarised in Table 1. Patients were recruited between 2013 and 2017 from 6 centres in NSW and Victoria, Australia, and data collection continued until March 2019, the last date of follow-up. The median time of follow-up post-transplant was 2.0 years (range: 0.1–5.0 years). This study was registered with the Australian Clinical Trials Registry (Registration number: ACTRN12613000487718). Ethics approval was obtained from the SCHN Ethics Committee (HREC/12/SCHN/441), and governance approval was also obtained from recruiting centres. All participants provided written informed consent.

**Table 1:** Patient characteristics, diagnosis and response parameters for 44 patients with myeloma who received HDM

***Patient characteristics: median (range)***	
Age (years) Weight (kg) BSA (m^2^) Creatinine clearance (ml/min/1.73m^2^)^a^ Haematocrit (L/L) Total melphalan dose (mg/m^2^)	60 (35–71) 83 (54–134) 2.0 (1.49–2.47) 88 (22–230) 0.34 (0.24–0.4) 200 (140–220)
***Sex (number of patients***	
males/females	31/13
***Immunologic isotype (number of patients)***	
IgG/IgA/Light chain	30/4/10
***International disease stage***	
1 / 2/ 3/ Unknown	21/15/6/2
***Maximum response pre HDM (number of patients)***	
Complete Very good partial Partial Minimal No change Not assessed	7 (of 40, 18%) 11 (of 40, 28%) 18 (of 40, 45%) 3 (of 40, 8%) 1 (of 40, 3%) 4
***Maximum response post HDM (number of patients)***	
Complete Very good partial Partial Minimal No change Not assessed	7 (of 38, 19%) 15 (of 38, 39%) 15 (of 38, 39%) 0 1 (of 38, 3%) 6

^a^Calculated using the Cockcroft and Gault equation [33]

Total melphalan dose = sum of test dose and high dose

### Melphalan test and subsequent dose administration and PK blood sampling

The first four patients received an intravenous (IV) test dose of 10 mg/m^2^ melphalan (Alkeran^®^, Aspen Pharmcare Australia Pty Ltd, NSW, Australia). Due to difficulties in detecting low melphalan concentrations with the available assay at this dose, we increased the test dose to 20 mg/m^2^ for the subsequent 40 patients. Levels were measured using high-performance liquid chromatography with ultraviolet detection (HPLC–UV) with detection and quantification limits of 0.1 µg/ml and 0.5 µg/ml, respectively, a linear range of 0.5 to 40 µg/ml and acceptable inter-day precision (< 6%) and accuracy (< 2% deviation from true concentration) for concentrations within the linear range [25].

The melphalan test dose (10 or 20 mg/m^2^), based on actual patient weight with no dose adjustment in obese patients, was administered 1–3 days prior to HDM administration and autograft. The HDM mg/m^2^ dose was either the remaining 190 or 180 mg/m^2^ (for a 200 mg/m^2^ total dose) or a modified dose at physician discretion (with a total dose permitted to be no higher than 220 mg/m^2^). The latter included HDM adjustments for patients with renal impairment. Both test and high melphalan doses were administered within 60 min of reconstitution, aiming for complete administration time within 90 min, as recommended in the Australian prescribing information [21]. Test and high-dose infusion concentrations were determined by dividing the administered dose (mg) by the infusion volume. Test and HDM infusion times were not standardised in the protocol, so data on the exact time from preparation to dose administration was retrospectively collected for 31 patients and allowed calculation of the time interval between dose reconstitution to infusion end.

Lithium heparin blood samples (2–4 ml) were collected after both test and subsequent doses via peripheral cannula at 5, 15, 30, 40, 75 and 150 min after completion of the infusions for measurement of plasma melphalan concentrations. The blood sampling strategy was selected based on our previously published limited sampling schedule [14]. For 5 (of 6) participating centres within NSW located 20 to 110 km from the PK Laboratory, the blood samples collected after the test dose were sent on dry ice on the same day as a collection to allow reporting of melphalan AUC results and dose recommendations in time for potential dose adjustments for the subsequent melphalan prescription on day-1, the day preceding autologous stem cell reinfusion. The remaining centre, 850 km remote, sent samples collected after the test and subsequent doses in a single batch for testing non-urgently.

### PK predictions of test and high dose Melphalan AUC using the trapezoidal rule

The PK software, Kinetica version 4.0, was used to calculate the test dose melphalan AUC using the trapezoidal rule. Predictions of the AUC for the subsequent high dose (AUC_pred_) were calculated assuming linear pharmacokinetics [17], by multiplying test dose AUC by the ratio of high dose (mg) to test dose (mg). For those institutions participating in real-time dose adjustment, the predicted dose required to achieve an AUC_cum_ of 12.8 mg/L.h, were communicated to the treating physician and the study clinical principal investigator in time for adjustment of the HDM, in addition to the values of AUC_pred_ for the remaining 190 or 180 mg/m^2^ doses (for the 10 and 20 mg/m^2^ test doses, respectively). Per protocol, the treating physician made the final decision regarding if and what dose adjustments were appropriate for the patient as long as the cumulative dose was no higher than 220 mg/m^2^, the highest surface-area-based dose previously shown to be safe [26]. Melphalan AUC was also measured after the subsequent dose (AUC_obs_) and compared with the AUC extrapolated from the test dose (AUC_pred_) using the paired Wilcoxon signed-ranks test. The bias and imprecision of the predictions were assessed as described below.

### Evaluation of predictive performance

Predictive performance was evaluated as previously described [27, 28]. Prediction error (PE) was first calculated using the equation PE = observed (*obs*)–predicted (*pred*) observation, then expressed as a percentage of the observed value; PE% = (*obs–pred*)/*obs* *100). Absolute prediction error (APE) was determined as │PE│. Mean prediction error (MPE) and 95% CI were also recorded as an estimate of bias. Root mean square prediction error (RMSE), calculated as $\mathrm{RMSE }= \sqrt{\frac{1}{n}{\sum \left(\frac{obs-pred}{pred}\right)}^{2}}$, then expressed as a percentage (RMSE%), provided an estimate of precision. Mean absolute prediction error (MAPE) was the mean of │PE│. The differences between observed and predicted observations were also evaluated by generation of a scatterplot of observed versus the ratio of predicted/observed observation as previously described [27, 28], and the fraction of ratios falling within a range of 0.8 to 1.2 was also calculated. All manipulation of data and statistical analyses were performed using SPSS version 25 or Microsoft Excel (Microsoft office 2010).

### Evaluation of factors with the potential to contribute to prediction error

This evaluation was conducted in the subgroup of patients who received the 20 mg/m^2^ test dose. Firstly, the paired Wilcoxin rank sign test was used to compare the following medication-related parameters related to test and high dose administrations: melphalan infusion concentration, infusion volume, infusion duration and time from dose preparation to infusion end. Secondly, prediction error in AUC was subdivided into two groups of PE% within 15% observed AUC, and PE% > ± 15% and the Mann Whitney test were used to identify whether there were significant differences between the two groups in infusion concentration or in creatinine clearance, fat-free mass and haematocrit. Thirdly, the Mann–Whitney test was used to compare the difference in PE% and APE for (1) patients recruited to the sites that undertook real-time PK assessment (*n* = 31) versus those recruited to the site that did not (*n* = 9) and (2) patients who received the melphalan test dose within 15 min (*n* = 5) versus the remainder (*n* = 22) and those who received the melphalan test dose within 30 min (*n* = 17) and the remainder (*n* = 10).

### PopPK Bayesian-based predictions of test and high dose Melphalan AUC

An external validation of the published PopPK model of HDM [14] was first performed using prediction-based diagnostic criteria as described in the Supplementary Appendix 1. This confirmed the reliability of the posterior Bayesian estimates of test and high dose clearance for a subsequent retrospective evaluation of PopPK Bayesian-based predictions of test and HDM AUC. The published PopPK model of HDM was reconstructed as previously described [14] using pre-test and HDM values for creatinine clearance, fat-free mass and haematocrit. The PK parameters were set to the published values shown in Table 2 using NONMEM 7.4 (Icon Development Solutions, Ellicott City, MD) with Perl-speaks-NONMEM library (version 4.9.0) and Pirana (version 2.9.9) as a graphical user interface. Posterior Bayesian estimates of melphalan clearance (CL) were obtained for the test and high doses for each patient and allowed calculation of individual estimates of AUC using the equation AUC = dose/CL. The accuracy of test dose PopPK Bayesian-based predictions of HDM exposure was examined in the same manner as used for the trapezoidal rule. The proportion of patients who achieved AUC_cum_ below, within and above the range of 10.9 to 14.7 mg/L.h using PopPK was also evaluated. This range represents the median AUC of 12.8 mg/L.h ± 15% from our previous published study [14].

**Table 2:** Summary of published PopPK model for HDM [14]

**PK parameter**	**Structural model**	**BSV (CV%)**	**Residual error**
CL (Lh)	CL = CL_NR_ + CL_R_ $CL_{{{\text{NR}}}}^{{}} = 17 \times \left( {HCT/34} \right)^{0.462} \times \left( {FFM/50} \right)^{0.75}$ $CL_{{\text{R}}} = 11.1 \times \left( {CL_{CR} /88} \right)$	26.**7**	*Ꝺ*_*1*_ = 0.072 *Ꝺ*_*2*_ = 0.082
V_1_ (L)	*V*_*1*_ = 13.2*(FFM/50)	57.9	
Q (L/h)	*Q* = 30.6	41.1	
V_2_ (L)	*V*_*2*_ = 15	34.5	

*CL* clearance, *V*_*1*_ volume of distribution into the central compartment, *Q* inter-compartmental clearance, *V*_*2*_ volume of distribution into the peripheral compartment, *BSV* between subject variability, *HCT* haematocrit (%), *FFM* fat free mass (kg) [34], *CL*_*CR*_ estimated creatinine clearance (mL/min/70 kg) [33], %*CV* coefficient of variation

### Assessment of disease response and transplant toxicity

Disease response criteria conformed to the 2011 Consensus Recommendation of the International Myeloma Working Group [29]. Immunoglobulin levels, serum electrophoretogram, immunofixation, serum free light chains and paraprotein quantitation were recorded: at diagnosis, within 7 days prior to ASCT; at 6 weeks (42 to 49 days), then 6, 12, 18 and 24 months post ASCT; and in the event of disease progression or re-initiation of therapy. The myeloma stage was recorded using both the Durie-Salmon and International Staging Systems [30, 31].

Records were kept of stem cell dose infused (CD34^+^ cells × 10^6^/kg), transfusion requirements and engraftment parameters and whether or not the patient received maintenance therapy (e.g. glucorticosteroids or thalidomide) post-HDM. Noninfectious or gastrointestinal toxicities (clinical oral mucositis, nausea, vomiting, diarrhoea, colitis) were recorded daily for all patients from day 2 to day 28 post-HDM using the National Cancer Institute Common Terminology Criteria for Adverse Events (Version 3). Other serious adverse effects, including febrile neutropenia, were also recorded.

## Results

### Patient demographics and pre-transplant characteristics

Baseline patient characteristics are shown in Table 1. Four (of 44, 9%) underwent HDM for relapsed or progressive disease. Pre-transplant chemotherapy-based induction regimens included cyclophosphamide, bortezomib and dexamethasone (*n* = 42), bortezomib and cyclophosphamide (*n* = 1) and dexamethasone and lenalidomide (*n* = 1). The best response to induction therapy at the time of HDM, including those with progressive disease, was complete response in 7 patients (18%), very good partial response in 11 (28%), partial response in 18 (45%), minimal response in 3 (8%) and stable disease in 1 (2%) of patients. This data was missing for 4 patients.

### Predictivity of test dose PK

The 10 mg/m^2^ test dose was associated with low measured concentrations and poor prediction accuracy of high dose AUC_pred_ in 2 (of 4) patients (PE% was > 59%). PK predictions from the increased 20 mg/m^2^ test dose are presented in Table 3. In these forty patients, the test dose ranged from 30–50 mg and was infused over 9–36 min in a volume of 57–250 ml. The subsequent high dose ranged from 220–450 mg and was infused over 15–45 min in a volume of 110–597 ml.

**Table 3:** Observed melphalan exposure (AUC_obs_) after test (20 mg/m^2^) and subsequent high dose and comparison with the AUC extrapolated from test dose (AUC_pred_) using the trapezoidal Rule and PopPK assessment methods, assuming linear pharmacokinetics

			**PK assessment using the trapezoidal rule**			**PK assessment using PopPK**		
Patient no	**Test dose mg/m**^**2**^**, (mg)**	**High dose mg/m**^**2**^** (mg)**	**Test dose AUC**_**obs**_** (mg/L.h)**	**HDM AUC**_**pred,**_** AUC**_**obs,**_** (PE%) (mg/L.h)**	**AUC**_**cum**_** (mg/L.h)**	**Test dose AUC**_**obs**_** (mg/L.h)**	**HDM AUC**_**pred,**_** AUC**_**obs,**_** (PE%) (mg/L.h)**	**AUC**_**cum**_** mg/L.h)**
1	20 (41)	180 (371)	1.35	12.2, 9.4 **(−29%)**	10.8	1.48	13.4, 10.3 **(−30%)**	11.8
2	20 (38)	180 (340)	1.35	12.1. 10.8 (**−**12%)	12.2	1.46	13.1, 12.1 (**−**8%)	13.6
3	20 (41)	180 (367)	1.32	11.8, 12.4 (5%)	13.8	1.30	11.6, 13.6 (14%)	14.9
4	20 (37)	180 (329)	1.72	15.3, 14.1 (**−**8%)	15.8	2.01	17.9, 15.1 **(−18%)**	17.1
5	20 (35)	180 (325)	1.12	10.4, 9.5 (**−**10%)	10.6	1.29	12.0, 10.5 (**−**14%)	11.8
6	20 (50)	180 (450)	0.99	8.9, 7.8 (**−**14%)	8.8	1.02	9.2, 8.7 (**−**6%)	9.7
7	20 (42)	180 (378)	1.32	11.9, 9.3 (**−27%**)	10.7	1.33	12.0, 10.5 (**−**15%)	11.8
8	20 (43)	180 (388)	1.22	11.0, 9.4 **(−17%)**	10.6	1.32	11.9, 10.7 (**−**12%)	12.0
9	20 (41.8)	180 (378)	1.64	14.8, 12.1 **(−22%**)	13.8	1.71	15.5,13.5 (**−**15%)	15.2
10	20 (36)	180 (320)	1.19	10.6, 11.8 (10%)	13.0	1.34	11.9, 11.7 (**−**2%)	13.0
11	20 (50)	180 (360)	1.28	11.5, 12.5 (8%)	13.8	1.32	11.9, 13.3 (11%)	14.6
12	20 (45)	180 (390)	1.27	11.0, 10.4 (**−**6%)	11.7	1.31	11.4, 11.2 (**−**2%)	12.5
13	20 (30)	180 (268)	1.32	11.8, 7.4 **(−60%)**	8.7	1.42	12.7, 8.2 **(−55%)**	9.6
14	20 (36)	180 (320)	1.22	10.8, 9.2 **(−16%**)	10.6	1.40	12.4, 10.0 **(−24%)**	11.4
15	20 (50)	180 (430)	1.13	9.7, 9.2 (**−**6%)	10.3	1.24	10.7, 10.6 (0%)	11.9
16	20 (45)	180 (400)	1.61	14.3, 12.9 (**−**11%)	12.9	1.72	15.3, 13.9 (**−**10%)	15.6
17	20 (40)	180 (350)	1.80	15.8, 16 (2%)	17.8	1.87	16.4, 16.7 (2%)	18.5
18	20 (45)	180 (395)	1.68	14.7, 12.4 **(−19%)**	14.1	1.71	15.0, 14.4 (**−**4%)	16.1
19	20 (40)	180 (340)	1.61	13.7, 11.9 (**−**15%)	13.8	1.58	13.4, 13.1 (**−**3%)	14.6
20	20 (40)	180 (360)	1.10	9.9, 11.0 (10%)	12.1	1.16	10.4, 10.4 (0%)	11.6
21	20 (36)	180 (330)	2.03	18.6, 13.2 **(−41%)**	13.2	1.87	17.1, 14.4 **(−19%)**	16.2
22	20 (42)	180 (380)	1.28	11.6, 10.1 (**−**14%)	11.4	1.37	12.4, 9.1 **(−37%)**	10.4
23	20 (39)	180 (350)	1.91	17.1, 14.8 **(−16%)**	16.7	1.91	17.1, 13.4 **(−28%)**	15.3
24	20 (40)	180 (370)	0.98	9.1, 9.9 (8%)	10.9	1.19	11.0, 10.4 (**−**6%)	11.6
25	20 (35)	180 (325)	1.41	13.1, 12.5 (**−**5%)	13.9	1.53	14.2, 12.7 (**−**12%)	14.3
26	20 (45)	180 (390)	1.68	14.6, 10.5 **(−39%)**	12.1	2.0	17.3, 15.1 (**−**15%)	17.1
27	20 (40)	180 (380)	1.02	9.7, 9.6 (**−**1%)	10.6	1.21	11.5, 10.8 (**−**6%)	12.0
28	20 (45)	180 (425)	1.30	12.3, 12.0 (**−**2%)	13.3	1.38	13.0, 12.2 (**−**7%)	13.6
29	20 (40)	180 (335)	1.77	14.8, 11.3 **(−32%)**	13.0	1.74	14.6, 11.3 **(−29%)**	13.0
30	20 (40)	180 (380)	1.44	13.7, 12.5 (**−**9%)	13.9	1.53	14.5, 13.2 (**−**10%)	14.8
31	20 (40)	180 (360)	1.66	14.9, 12.6 **(−19%)**	14.2	1.69	15.2, 14.0 (**−**8%)	15.7
32	20 (35)	180 (320)	1.20	11.0, 11.7 (6%)	12.9	1.29	11.8, 13.2 (10%)	14.5
33	20 (44)	180 (400)	1.44	13.1, 9.0 **(−45%)**	10.5	1.31	11.9, 7.9 **(−50%)**	9.3
34	20 (35)	180 (300)	1.25	10.7, 6.3 **(−69%)**	7.6	1.48	12.7, 7.4 **(−71%)**	8.9
35	20 (37.5)	180 (335)	1.16	10.4, 10.6 (2%)	11.8	1.33	11.9, 11.3 (**−**6%)	12.6
36	20 (39)	186* (360)	1.22	11.3, 10.5 (**−**7%)	11.7	1.50	13.8, 12.0 **(16%)**	13.5
37	20 (43)	188* (400)	1.02	9.5, 10.4 (8%)	11.4	1.18	11.0, 10.9 (0%)	12.1
38	20 (36.5)	120* (220)	2.64	15.9, 14.5 (**−**10%)	17.1	2.43	14.6, 14.5 (**−**1%)	17.0
39	20 (40)	200* (400)	0.83	8.3, 8.3 (0.0%)	9.2	1.07	10.7, 9.8 (**−**9%)	10.9
40	20 (45)	200* (430)	1.23	11.8, 9.8 **(−20%)**	11.1	1.44	13.8, 11.1 **(−24%)**	12.6

^*^Patients received an adjusted high dose based on the PK results from the test dose

Trap = AUC determined using trapezoidal rule

PopPK = AUC determined from Bayesian forecasting using a previously published PopPK model for HDM [14]

PE% = (AUC_Obs_–AUC_Pred_)/AUC_Obs_ *100

AUC_cum_ = sum of AUC_obs_ for test and high doses

Using the trapezoidal rule, test dose AUC_obs_ was mean (95%CI) 1.39 (0.98–2.06) mg/L.h. For the high dose, AUC_pred_ was significantly higher than AUC_obs_ using the paired Wilcoxon signed-ranks test: median 12.3 (range: 8.9, 17.2) versus 11.0 (7.4, 14.9) mg/L.h, (*p* < 0.001). Significant bias was observed in the high dose AUC predictions since the PE% of mean (95%CI): − 14% (− 60%, 10%) was significantly different to zero using the one sample T-Test (*p* < 0.001). The APE of the mean (95%CI): 2.0 (0.03–6.35) mg/L.h was also significantly different to zero using the one sample T-Test (*p* < 0.001). High dose AUC_pred_ was within ± 15% AUC_obs_ for 25/40 (63%) patients.

### Feasibility of providing real-time result to treating physician

Five centres within NSW, Australia, located 20 to 110 km from the PK laboratory, were able to transport samples by the morning after the collection date, and all samples arrived frozen. PK results for the test dose (20 mg/m^2^) were available to the treating physician in time for all 31 patients enabling possible adjustment of the subsequent HDM. Of the 31 patients included in this subgroup, 26 were administered HDM 180 mg/m^2^ providing a total dose of 200 mg/m^2^ (Table 3, patient numbers 1 to 35) and the median (range) for AUC_pred_, AUC_obs_ and AUC_cum_ were 11.85 (8.91–18.6) mg/L.h, 10.9 (7.4–16) mg/L.h and 12.2 (8.7–17.8) mg/L.h, respectively. Five patients received an adjusted dose that was based on the PK results (total dose range 140–220 mg/m^2^) and their individual results are shown in Table 3 (patient numbers 36 to 40). Four of these 5 patients had dose increases in response to low AUC_pred_ results ranging from 8.3 to 11.75 mg/L.h (AUC_obs_ ranged from 8.3 to 10.5 mg/L.h). The remaining patient (number 38) had impaired renal function and with a relatively high AUC_pred_ value of 15.91 mg/L.h, received a reduced HDM dose of 120 mg/m^2^ to provide a total dose of 140 mg/m^2^. The AUC_obs_ and AUC_cum_ values for this patient were 14.5 and 17.1, respectively. AUC predictions were within 15% in 80% (4 of 5) patients who received the adjusted HDM.

### Evaluation of factors with the potential to contribute to prediction error

Melphalan infusion concentration for the test dose (median, range: 0.17, 0.12–0.76 mg/ml) was significantly lower than that of the high dose (median, range: 1.52, 0.56–3.15 mg/ml), *p* < 0.001, and there were also significant differences in infusion volume, infusion duration and time interval from dose preparation to infusion end (Table 4). Test and high dose infusion concentrations were lower in patients with large (> ± 15%) prediction error in AUC, but the differences were not significant (*p* = 0.078, 0.228, respectively). Creatinine clearance, fat-free mass and haematocrit were not significantly associated with large prediction error.

**Table 4:** Comparison of test and high dose preparation and administration parameters

	**Test Dose**	**High dose**	**Significance**^**a**^
Infusion concentration (mg/ml)	0.17 (0.12–0.76)	1.52 (0.56–3.15)	*P* = 0.005
Infusion volume (ml)	250 (57–250)	250 (110–597)	*P* = 0.000
Infusion duration (min)	14.4 (9–36)	28.2 (15–45)	*P* = 0.000
Time from dose preparation to infusion end (min)	51.8 (21.8–96.0) (*n* = 27)	68.0 (34.8–92.0) (*n* = 27)	*P* = 0.000
Time from dose preparation to infusion start (min)	25 (7–60) (*n* = 27)	35 (14–60) (*n* = 27)	*P* = 0.011

Data are median (range)

^a^Paired Wilcoxin signed-rank test

The time interval from melphalan dose preparation to infusion end was significantly lower for the test dose compared with the high dose: median (range) was 51.8 (21.8–96) min versus 68 (34.8–92) min, *p* < 0.001 (Table 4). Patients who received the test dose within 15 min of reconstitution had significantly lower prediction error in AUC values than those who received the test dose greater than 15 min (and less than 60 min) after reconstitution: the median (range) for PE% was 3.2% (−10%, 18%) versus −16% (−67%, 26%), *p* < 0.05, and APE was median (range): 1.19 (0.36–2.18) mg/L.h versus 1.68 (0.21–6.38), *p* = NS. When the test dose administration time was 16–60 min, accuracy was within 15% in 59% of patients and improved to 80% when the time was reduced to less than 15 min. No significant differences in PE% or APE were observed when a 30 min cut-off for test dose administration time was used. High dose administration time was also not associated with significantly altered prediction accuracy. Participation in real-time PK assessment was not found to significantly impact on the magnitude of prediction error: median (range) for PE% was −12% (−60%, 10%) for the 31 patients who participated in real-time PK monitoring and −17% (−67%, 26%) for those that did not (*n* = 9).

### Evaluation of the predictive capacity of the published PopPK model for HDM in myeloma

The external validation results for the published PopPK model for melphalan [14], presented in Supplementary Appendix 1 and Supplementary Figs. 1 and 2, were found to be within acceptable limits (within ± 25%) for both the test and high doses when individual concentration data and Bayesian forecasting was included with the population data, providing confidence in its predictive capacity for doses ranging from 10 to 220 mg/m^2^.

When the PopPK model was used to derive PK predictions from the 20 mg/m^2^ test melphalan dose, the AUC_obs_ values were mean (95% CI) 1.49 (1.07, 2.03) mg/L.h. For the high dose, PopPK estimates of AUC_pred_ were significantly higher than AUC_obs_ using the paired Wilcoxon signed-ranks test: 13.2 (10.4, 17.4) versus 11.8 (7.9, 15.2) mg/L.h, *p* < 0.001. PE% of mean (95%CI) − 13% (− 55%, 11%) was significantly different to zero using the one sample T-Test (*p* < 0.001) indicating significant bias. The APE of mean (95%CI): 1.63 (0–5.3) mg/L.h was also significantly different to zero using the one sample T-Test (*p* < 0.001). High dose AUC_pred_ was within ± 15% AUC_obs_ in 28/40 (70%) of patients.

### Cumulative exposure to melphalan

Total melphalan cumulative exposure for the 40 patients who received the 20 mg/m^2^ test dose was mean (95%CI): 12.4 (7.5, 17.8) mg/L.h using the trapezoidal rule and 13.3 (8.9, 18.5) mg/L.h using PopPK. Using PopPK 12% (5/40), 58% (23/40) and 30% (12/40) of patients had AUC_cum_ below, within and above the 10.9 to 14.7 mg/L.h range, respectively.

### Toxicity and outcome post-HDM

Patients received a median 3.62 × 10^6^/kg CD34^+^ cells (range, 1.72 to 17.99 × 10^6^/kg). The median time to neutrophil recovery to ≥ 0.5 × 10^9^/L was 11 days (range:3–23), and the median time to platelet recovery > 20 × 10^9^/L was also 11 days (range: 1–65, excluding 1 patient whose neutrophil and platelet counts did not drop below 1.5 × 10^9^/L and 23 × 10^9^/L, respectively. Twenty four/42 (57%) required red cell transfusion (median 3 units; range 1–8) and 38/42 (90%) required platelet transfusions (median 2 single donor equivalents; range 1–18). Seventy-three percent of patients (32/44) experienced febrile neutropenia. The incidence of NCTC clinical oral mucositis were grade 0 (15/41, 37%), grade 1 (11/41, 27%), grade 2 (7/41, 17%) and grade 3 (8/41, 19%). The incidence of other gastrointestinal toxicities ≥ grade 3 was 5% (2/41) for colitis, 12% (5/41) for nausea, 2% (1/41) for vomiting and 12% (4/41) for diarrhoea.

Overall response after induction therapy plus HDM was complete response in 7/38 (19%) patients, very good partial response in 15 (39%), partial response in 15 (39%) and stable disease in 1 (3%), including four patients being treated for progressive disease. The 100-day transplant-related mortality was 0%. After a median follow-up of 1.97 years (range: 0.09–4.96), 8 patients (19%) have died from disease progression (*n* = 5), sepsis (*n* = 1), cardiac (*n* = 1) and unknown causes (*n* = 1). At the time of the analysis, disease progression had occurred in 23/44 (52%) of patients.

## Discussion

With 40 patients, this is the largest study to date in which the feasibility and accuracy of AUC predictions of HDM actual AUC attainment from a 20 mg/m^2^ test dose were evaluated. At five centres within NSW, Australia (located from 20 to 110 km from the testing laboratory), test dose PK assessment in time for possible adjustment of the subsequent HDM was demonstrated to be logistically feasible. All samples arrived at the laboratory with good integrity indicating that there were no issues related to transportation and storage of plasma samples.

Within this pilot study, the clinicians caring for the patients had full control over the administered HDM dose, and, as such, the attainment of an HDM AUC within a specific target range was not the primary aim. The majority of patients received a further 180 mg/m^2^ HDM, and 21 of these achieved adequate HDM exposure above 10.9 mg/L.h, with the clinician being reassured that there was no need to modify the dose. Five patients continued to receive the 180 mg/m^2^ dose in spite of low AUC_pred_ results ranging from 8.9 to 10.6 mg/L.h which can be explained by the initial clinician reluctance to change the dose. On five occasions clinicians did utilize the option of modifying HDM in response to the PK results with excellent accuracy in 80% of cases, including the patient with impaired renal function for whom the suitability of the published dose recommendations in renal impairment [9] was confirmed by the PK results.

PK predictions of HDM AUC attainment from the 20 mg/m^2^ test dose were within 15% observed values in 63% of patients using the trapezoidal rule. There was a tendency for the test dose PK assessments to over-predict HDM exposure which was indicated by significant bias. This was likely related to the fact that delay times of between 16 and 60 min were not ideal for the lower melphalan concentrations of median 0.17 (range: 0.12–0.76) mg/ml administered in the test dose, which were likely affected by stability issues [21, 22] leading to a reduction in the administered dose. Published data has indicated that a melphalan infusion concentration of 0.5 mg/ml has limited stability of less than 1 h at room temperature [21, 22]. Moreover, test dose infusion durations varied, ranging from 9 to 36 min, and were only slightly reduced when compared with HDM (range: 15 to 45 min), and there may have been decomposition of the drug during the infusion. During the analysis, it was appreciated that the volume and rate of administration of the test dose, as well as the high dose, was under the control of the individual centres and had not been dictated by the protocol. If the protocol had specified the test and high dose infusion volumes (to obtain infusion concentrations of 2 or 4 mg/ml that are stable for up to 2 h and 8 h, respectively at room temperature [22]), and required similar infusion rates (in mg/m^2^/min) for the test and high doses (as has previously been applied in busulfan therapeutic monitoring [32]), then predication accuracy may well have been higher. These strategies together with the use of a higher “test” dose, such as an equal split in dose as has been used in some studies [19], should improve the stability of the melphalan infusions and the accuracy of the concentration measurements. It remains to be seen whether this provides more frequent achievement of the desired AUC. It should be noted, however, that many of our recommendations are contrary to the administration guidelines stated in the Australian product information for melphalan [21], which recommend infusion concentrations no greater than 0.45 mg/ml and infusion times of minimum of 15 min. This highlights the inadequacy of the recently revised (September 2021) Australian product information, which still does not include its current clinical usage at high doses.

Whilst the tendency for over-prediction of the high dose AUC observed in this study could lead to potential below-target dosing, it should be noted that more than 87% of patients achieved a posterior Bayesian estimate of total cumulative exposure above 10.9 mg/L.h. Moreover, there were no incidences of unexpected severe toxicity; the transplant-related mortality was zero and the incidences of ≥ grade 3 oral mucositis, and other gastro-intestinal toxicities were comparable to that previously observed [14, 15]. Our conservative approach to dose escalation, with a maximum allowable combined dose in the protocol being 220 mg/m^2^, meant that no patient was exposed to what is conventionally considered unsafe high levels of HDM. Indeed, whilst the protocol allowed for a total dose of 220 mg/m^2^, this was only adopted in two patients who received the 20 mg/m^2^ test dose; one of these who only achieved AUC_cum_ of 9.2 mg/L.h and may well have benefited from a higher dose.

The current dataset provided an external validation of the previous published PopPK model [14] for both the test and HDM, with prediction-based evaluations of melphalan concentrations showing improved predictive capability for estimates generated using Bayesian forecasting compared with population-based estimates, as is commonly observed [24, 27]. The Bayesian predictions of test dose concentrations were less accurate and precise than that for the HDM, but still within acceptable limits [24, 27], since the MPE% was < 5%, the RMSE % was ≤ 15% demonstrating the validity of applying the previously published PopPK model to both the test and HDM data sets, even though the test doses of 10 and 20 mg/m^2^ were outside the dose range evaluated in the original PopPK study [14]. When used to make PK predictions of HDM AUC attainment from the 20 mg/m^2^ test dose, predicted AUC values were within 15% observed values in 70% of patients, which was improved compared with the trapezoidal rule, and there was also slightly reduced bias in the predictions. The excellent predictive performance of this PopPK model suggests that it may now be used in the clinical setting for therapeutic monitoring purposes.

In conclusion, this pilot study has demonstrated that test dose prediction of actual HDM AUC attainment was accurate to within 15% in up to 70% of patients and has the potential to provide more consistent exposure to HDM, particularly if steps are taken to further improve prediction accuracy by optimising melphalan infusion preparation and administration. This melphalan PK study has highlighted the issues related to melphalan infusion instability, which, once addressed, will improve the feasibility of moving towards a patient-specific approach to optimise their myeloma response. HDM and ASCT remain central to the management of fit patients with myeloma. Such patients commit to a resource-intensive therapy with significant morbidity.

## Supplementary Information

Below is the link to the electronic supplementary material.

Supplementary file1 (DOCX 166 kb)

## Data Availability

The data generated as part of this study are available on request from the corresponding author following Human Research Ethics Committee approval. The data are not publicly available due to privacy and ethical restrictions.
