# Supplementary material for: Challenges associated with test dose pharmacokinetic predictions of high dose melphalan exposure in patients with multiple myeloma
Source: Eur J Clin Pharmacol. 2022 Oct 7;78(12):1911–21. doi: 10.1007/s00228-022-03396-x (PMC9649448; doi:10.1007/s00228-022-03396-x)

**Supplementary Information: European Journal of Clinical Pharmacology**

**CHALLENGES ASSOCIATED WITH TEST DOSE PHARMACOKINETIC PREDICTIONS OF HIGH DOSE MELPHALAN EXPOSURE IN PATIENTS WITH MULTIPLE MYELOMA.**

Christa Ellen Nath^1,2,9^ (corresponding author, email:christa.nath@health.nsw.gov.au, orcid i.d.0000-0002-1013-3646), Andrew Grigg^3^ ,Sebastian P.A. Rosser^1,2^(orcid i.d. 0000-0002-0203-4904), Jane Estell^4,9^, Elizabeth Newman^4^, Campbell Tiley^5^, Sundra Ramanathan^6^, Shir Jing Ho^6,10^ (orchid i.d. 000-0002-5368-8975),Stephen Larsen^7,9^, John Gibson^7,9^, Peter Presgrave^8^, Peter John Shaw^2,9^, Judith Trotman^4,9^

^1^ Biochemistry Department and ^2^Cancer Centre for Children, The Children’s Hospital at Westmead, Australia, ^3^ Haematology Department, Austin Health, Australia ^4^ Haematology Department, Concord Repatriation General Hospital, Australia, ^5^ Haematology Department, Gosford Hospital, Australia , ^6^ Haematology Department, St George Hospital, Australia ^7^ Haematology Department , Royal Prince Alfred Hospital, Australia, ^8^ Haematology Department, Wollongong Hospital, Australia, ^9^ Faculty of Health and Medicine, The University of Sydney, Australia,^10^ The University of New South Wales

**Evaluation of published PopPK model for HDM in myeloma patients**

***Methods:*** The predictive capability of our previously published PopPK model for HDM [14] developed in a cohort of 100 patients (aged 36-73 years) with myeloma, was first externally validated using prediction-based diagnostic criteria. The PK parameters were fixed to the final PopPK parameter estimates summarized in Table 2 and applied to the test and HDM data collected as part of the current study using the nonlinear mixed effects modelling software NONMEM 7.4 (Icon Development solutions, Ellicott city, MD) with Perl-speaks-NONMEM library (version 4.9.0) and Pirana (version 2.9.9) as a graphical user interface. Population-predicted melphalan concentrations (C_pred_) and posterior Bayesian estimates of the individual melphalan concentrations (C_ipred_) were generated for each observed concentration data point (C_obs_) in the test and HDM dose data sets and the predictive performance was evaluated as described in the manuscript.

***Results:*** Figures 1A/1B and 2A/2B are scatterplots of C_i/pred_/C_obs_ ratio versus C_obs_ for the Test and High melphalan doses, respectively. A higher proportion of ratios were within a range of 0.8 to 1.2 for individual Bayesian predictions compared with population-predictions: 91% and 99.6%, compared with 59% and 70%, for the test and high doses, respectively. Test dose MPE was 0.04 (95% CI -0.03, 0.16) µg/ml for C_ipred_ and 0.06 (95% CI: -0.19, 0.39) µg/ml for C_pred_. PE% was mean 4.1% (95%CI -32, 23%) for C_ipred_ and mean 5% (95%CI, -66, 47%) for C_pred_. RMSE was 15% for C_ipred_ and 34% for C_pred_.

High dose MPE was 0.21 (95% CI -0.27, 0.92) µg/ml for C_ipred_ and -0.02 (95% CI: -2.0, 2.2) µg/ml for C_pred_. PE% was mean 5.6% (95%CI -7, 14%) for C_ipred_ and mean -3.0% (95%CI, -61, 39%) for C_pred_. RMSE was 8% for C_ipred_ and 24% for C_pred_.

Figure 1 Scatterplots of Ratios of (A) individual and (B) population-predicted melphalan concentrations to observed concentration versus observed concentration following the test dose.


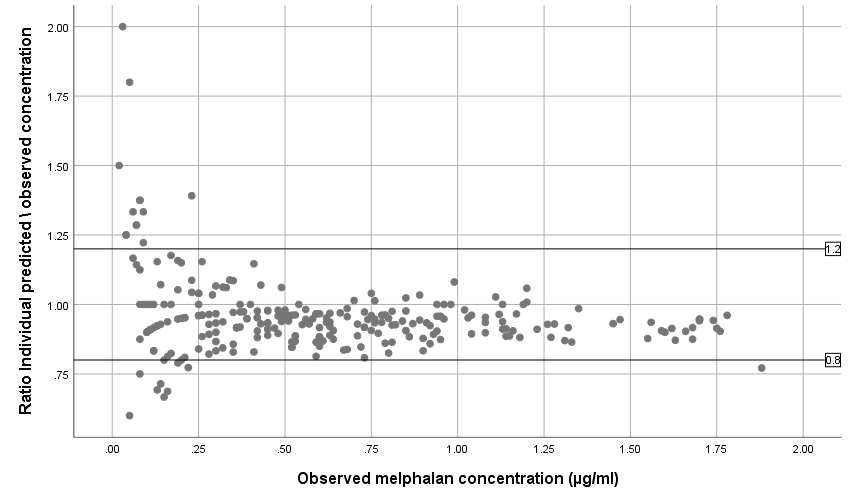


(B)


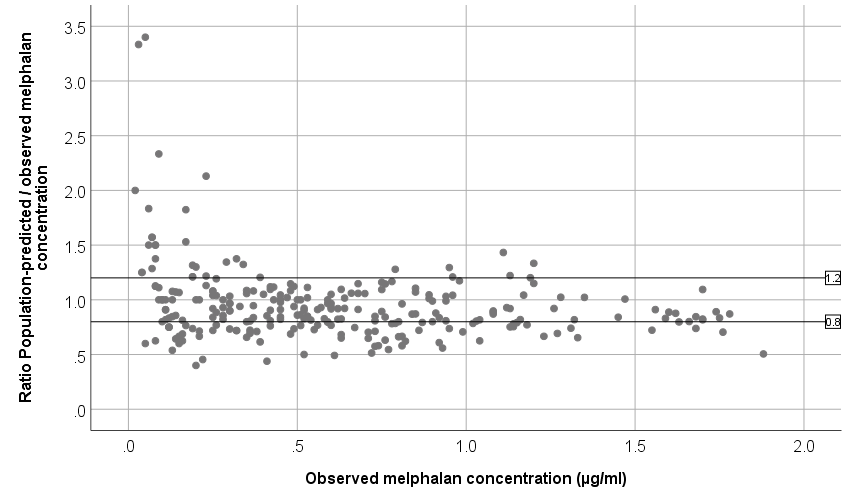


Figure 2. Scatterplots of Ratios of (A) individual and (B) population-predicted melphalan concentrations to observed concentration versus observed concentration following the high dose.


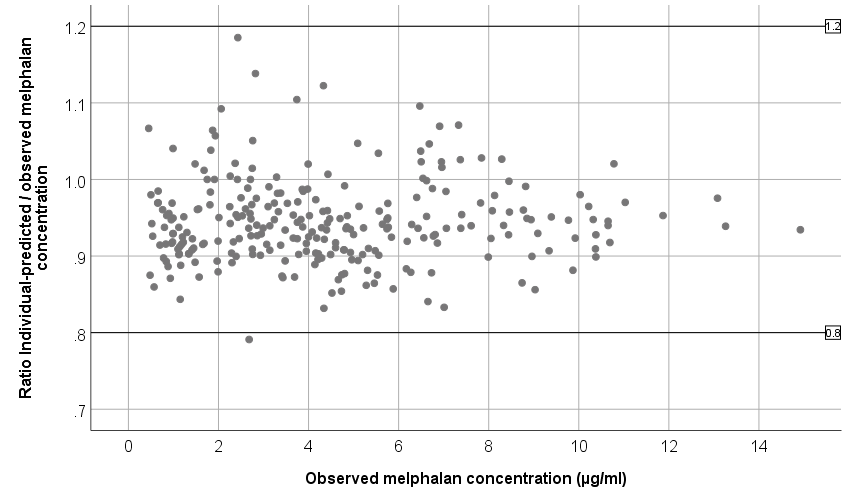


(B)


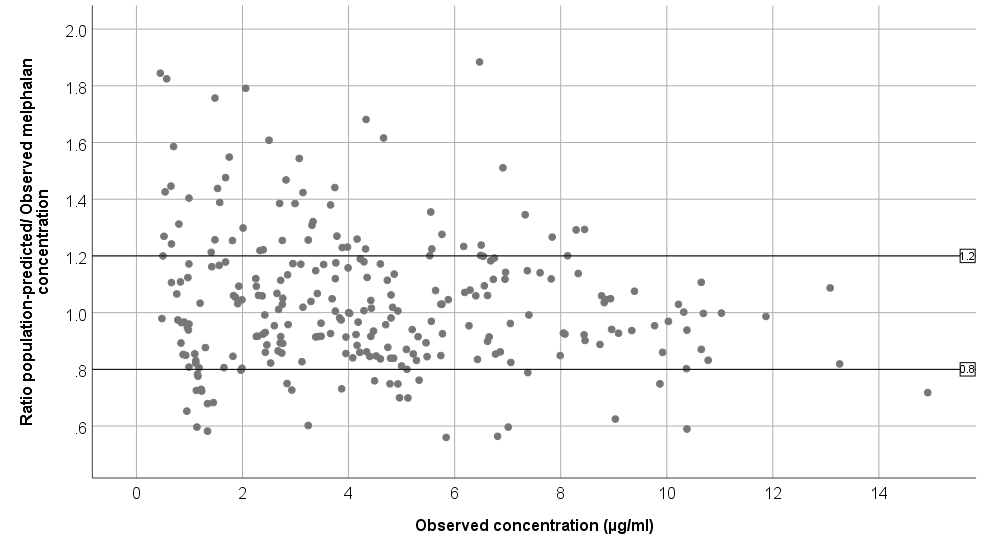

Supplement: Supplementary file 1 — Supplementary file1 (DOCX 166 kb) [file 228_2022_3396_MOESM1_ESM.docx]
